# Muscle-specific overexpression of AdipoR1 or AdipoR2 gives rise to common and discrete local effects whilst AdipoR2 promotes additional systemic effects

**DOI:** 10.1038/srep41792

**Published:** 2017-02-01

**Authors:** Sahar Keshvari, Darren C. Henstridge, Choaping Ng, Mark A. Febbraio, Jonathan P. Whitehead

**Affiliations:** 1 University of Queensland, Mater Research Institute-UQ, Brisbane, QLD 4102, Australia; 2 Cellular and Molecular Metabolism Laboratory, Baker IDI Heart and Diabetes Institute, Melbourne, VIC 3004, Australia; 3 Division of Diabetes & Metabolism, Garvan Institute of Medical Research, Darlinghurst, NSW 2010, Australia

## Abstract

Hypoadiponectinemia and adiponectin resistance are implicated in the aetiology of obesity-related cardiometabolic disorders, hence represent a potential therapeutic axis. Here we characterised the effects of *in vivo* electrotransfer-mediated overexpression of the adiponectin receptors, AdipoR1 or AdipoR2, into tibialis anterior muscle (TAM) of lean or obese mice. In lean mice, TAM-specific overexpression of AdipoR1 (^TAM^R1) or AdipoR2 (^TAM^R2) increased phosphorylation of AMPK, AKT and ERK and expression of the insulin responsive glucose transporter *glut4*. In contrast, only ^TAM^R2 increased *pparα* and a target gene *acox1*. These effects were decreased in obese mice despite no reduction in circulating adiponectin levels. ^TAM^R2 also increased expression of *adipoQ* in TAM of lean and obese mice. Furthermore, in obese mice ^TAM^R2 promoted systemic effects including; decreased weight gain; reduced epididymal fat mass and inflammation; increased epididymal *adipoQ* expression; increased circulating adiponectin. Collectively, these results demonstrate that AdipoR1 and AdipoR2 exhibit overlapping and distinct effects in skeletal muscle consistent with enhanced adiponectin sensitivity but these appear insufficient to ameliorate established obesity-induced adiponectin resistance. We also identify systemic effects upon ^TAM^R2 in obese mice and postulate these are mediated by altered myokine production. Further studies are warranted to investigate this possibility which may reveal novel therapeutic approaches.

Adiponectin is a key adipokine that displays a variety of beneficial effects to reduce diabetes, atherosclerosis and cardiometabolic disease^1^. Adiponectin regulates carbohydrate and lipid metabolism, reducing hepatic glucose production and enhancing fatty acid oxidation in skeletal muscle^1^. In contrast to most other adipokines, circulating adiponectin levels are typically reduced in obesity, type 2 diabetes and associated conditions^1^. Moreover, mice lacking adiponectin or humans with polymorphisms that compromise adiponectin production develop metabolic dysfunction and or type 2 diabetes^2^. Hence, therapeutic strategies to reverse hypoadiponectinaemia are attractive. Increasing evidence indicates that adiponectin resistance also contributes to the development of metabolic and cardiovascular diseases^3^,^4^,^5^,^6^,^7^,^8^,^9^,^10^,^11^. While the molecular mechanisms that give rise to adiponectin resistance are poorly defined strategies to overcome adiponectin resistance are also of therapeutic potential.

The beneficial effects of adiponectin are mediated primarily via the action of two atypical seven-transmembrane domain (7TMD) receptors, AdipoR1 and AdipoR2^12^. These receptors are structurally and functionally distinct from other 7TMD receptors, having intracellular N-termini and extracellular C-termini, and couple adiponectin to a range of downstream effectors including AMPK, PPARα, AKT and ERK by mechanisms that are incompletely understood^12^,^13^,^14^,^15^. Molecular and cellular studies have revealed differences between AdipoR1 and AdipoR2 that include different binding properties^12^, cell surface expression^16^,^17^,^18^ and temporal signaling profiles^19^,^20^. Furthermore, investigations in mice have demonstrated contrasting expression profiles, with AdipoR1 expressed relatively ubiquitously compared with AdipoR2^12^, and activation of alternate signaling pathways^21^. For example, studies in knockout mice indicate that in liver activation of AMPK is mediated primarily by AdipoR1 whilst PPARα appears to be downstream of AdipoR2^21^.

In the current study we aimed to extend our cell-based investigations demonstrating differences between AdipoR1 and AdipoR2^17^,^20^ to compare the effects of overexpression of either AdipoR1 or AdipoR2 in skeletal muscle in lean and obese mice. Overexpression was achieved by *in vivo* electrotransfer (IVE) of the tibialis anterior muscle (TAM) that allowed characterization of local, TAM-specific changes in phosphorylation of downstream signaling effectors and expression of genes involved in glucose and lipid metabolism as well as determination of somewhat unexpected systemic effects in response to overexpression of AdipoR2.

## Results

### Overexpression of AdipoR2 in TAM of obese mice has unexpected systemic effects

Using *in vitro*, cell-based systems we previously demonstrated that acute treatment with globular adiponectin resulted in different temporal signaling profiles in cells overexpressing AdipoR1 or AdipoR2, with the former promoting relatively acute activation (peaking at 15 min) and the latter more chronic activation (peaking after 24 h) respectively^20^. In the present study we have employed *in vivo* electrotransfer (IVE) to extend these observations and determine the effects of short-term (14 day) overexpression of AdipoR1 and AdipoR2 in mouse tibialis anterior muscle (TAM) of lean (chow fed) and obese mice fed a high fat diet (HFD) for 8 weeks.

**Figure 1:**
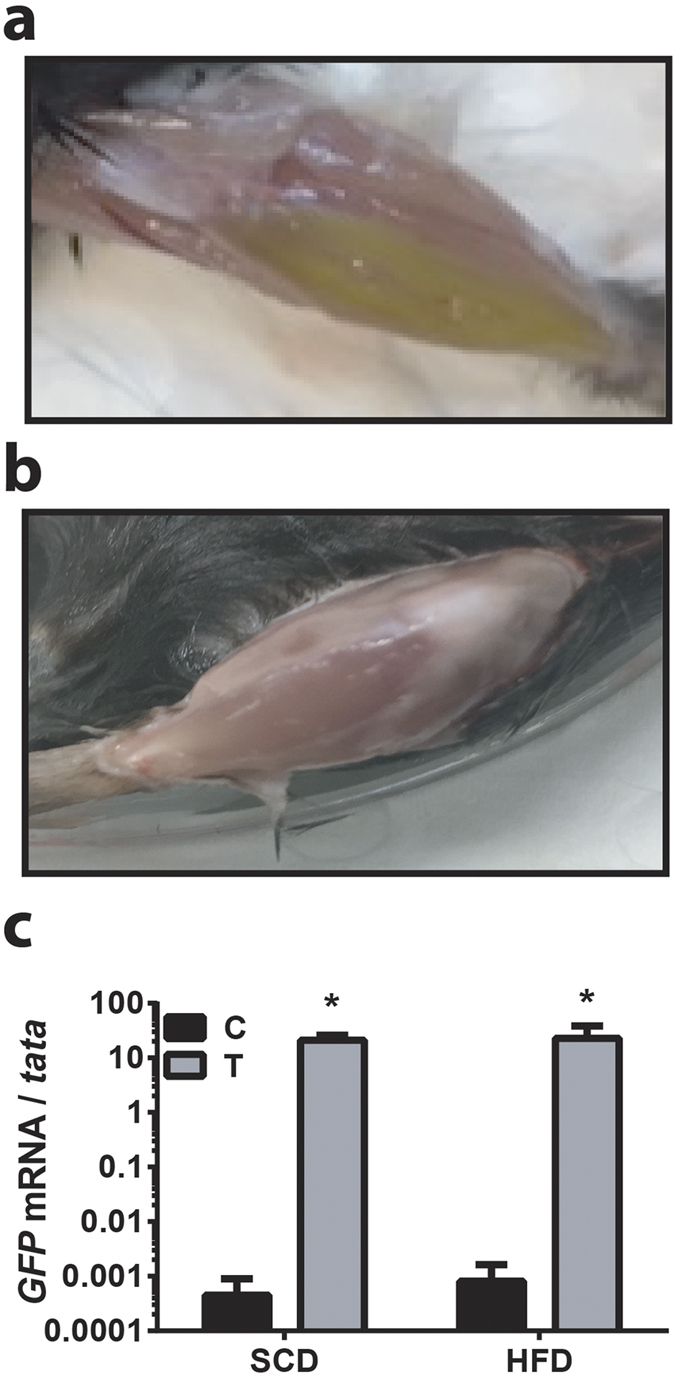
IVE-mediated expression of GFP. GFP protein expression in **(a)** test and **(b)** control (empty vector) TAM at the time of tissue collection. **(c)** qRT-PCR analysis showing *GFP* expression in test (T) verses control (C) TAM in lean and obese mice. Data are expressed as mean ± SEM; n = 3 in each group; *p < 0.05.

IVE is a powerful experimental approach that allows manipulation of the gene of interest in the test leg and comparison with the control leg in the same animal^22^,^23^. Here, we first used IVE to introduce a plasmid encoding GFP into the right TAM (test) and empty plasmid into the left TAM (control) of 16 week old lean or obese C57BL/6 mice. After 14 days mice were sacrificed and GFP expression was examined visually and by qRT-PCR. Visual inspection (under standard laboratory lighting) revealed robust GFP expression throughout the entire target muscle and none in neighbouring muscles or in the control leg in the same animal (Fig. 1a and b). GFP expression in the test TAM, but not the control TAM, was also confirmed by qRT-PCR (Fig. 1c).

Having confirmed the efficacy and specificity of the IVE protocol we then went on to perform experiments to characterise the effects of TAM-specific overexpression of AdipoR1 (^TAM^R1) or AdipoR2 (^TAM^R2) in lean (average body weight 29.85 g) and obese mice (average body weight 40.75 g). Following IVE of AdipoR1 or AdipoR2 into the test TAM and empty plasmid into the control TAM mice were maintained on chow or HFD for a further 14 days then sacrificed for analysis. To our surprise in obese mice ^TAM^R2 resulted in significantly reduced weight gain and elevated circulating adiponectin levels compared with ^TAM^R1 obese mice (weight gain: −0.6 ± 0.3 vs 1.3 ± 0.3 g, p = 0.001; adiponectin: 3.0 ± 0.3 vs 1.8 ± 0.2 μg/ml, p < 0.01; n = 6/group).

### HFD induced obesity does not alter AdipoR1 or AdipoR2 expression in TAM

**Figure 2:**
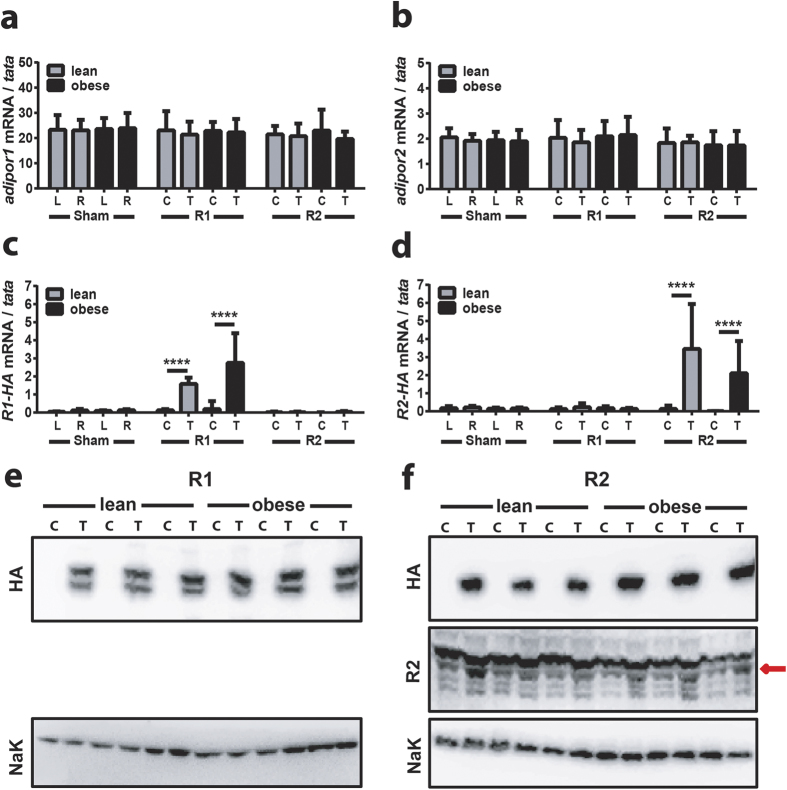
Characterisation of IVE-mediated overexpression of AdipoR1 and AdipoR2. qRT-PCR analysis of expression levels of endogenous mouse **(a)** *adipoR1* and **(b)** *adipoR2* and exogenous human **(c)** *R1-HA* and **(d)** *R2-HA* expression in left vs right TAM of control (sham) and test (T) vs control (C) TAM of ^TAM^R1 (R1) and ^TAM^R2 (R2) mice. Data are expressed as mean ± SEM; n = 6 in each group; ****p < 0.0001. **(e)** Representative western blot of HSP (membrane fractions) of TAM lysates derived from ^TAM^R1 mice probed with HA antibody (top panel) and NaK ATPase antibody (lower panel). **(f)** Representative western blot of HSP (membrane fractions) of TAM lysates derived from ^TAM^R2 mice probed with HA antibody (top panel), in-house R2 antibody (middle panel) and NaK ATPase antibody (lower panel); n = 3 in each group of lean and obese mice.

The effects on weight gain and circulating adiponectin levels were not anticipated and prompted us to redesign the study to include lean and obese control (sham) groups that were transduced with empty plasmid in both the left and right TAMs in parallel to the AdipoR1 or AdipoR2 transduced mice to allow direct comparison across groups. The extent of ^TAM^R1 or ^TAM^R2 was determined 14 days post IVE. Expression of both endogenous and exogenous genes was determined using primers specific for either mouse or human receptors respectively. The expression of endogenous receptors was unaffected by either IVE or diet (Fig. 2a and b), with *adipoR1* expressed at levels 10-fold higher than *adipoR2*. Human *AdipoR1* and *AdipoR2* were only detected in the test TAM and were expressed at similar levels in lean and obese mice (Fig. 2c and d). Western blot analysis was performed to characterise overexpression at the protein level. Western blot using HA-antibody confirmed expression of the exogenous HA-tagged proteins in only the test TAM and demonstrated that AdipoR1 and AdipoR2 proteins were expressed at similar levels in lean and obese mice (Fig. 2e and f). Western blot with a validated AdipoR2 antibody (that recognises both human and mouse AdipoR2^24^) revealed a 2-fold increase in total AdipoR2 in TAM from the test leg compared to the control leg. Unfortunately we were unable to perform a similar analysis of AdipoR1 protein due to the lack of a suitable, validated antibody that recognised both human and mouse AdipoR1 efficiently. Nevertheless, these results demonstrated the success and efficiency of the IVE approach and also showed that expression of the endogenous or exogenous receptors was not affected by HFD-induced obesity.

### ^TAM^R1 or ^TAM^R2 promotes activation of proximal signaling events and these effects are reduced in HFD-induced obesity

**Figure 3:**
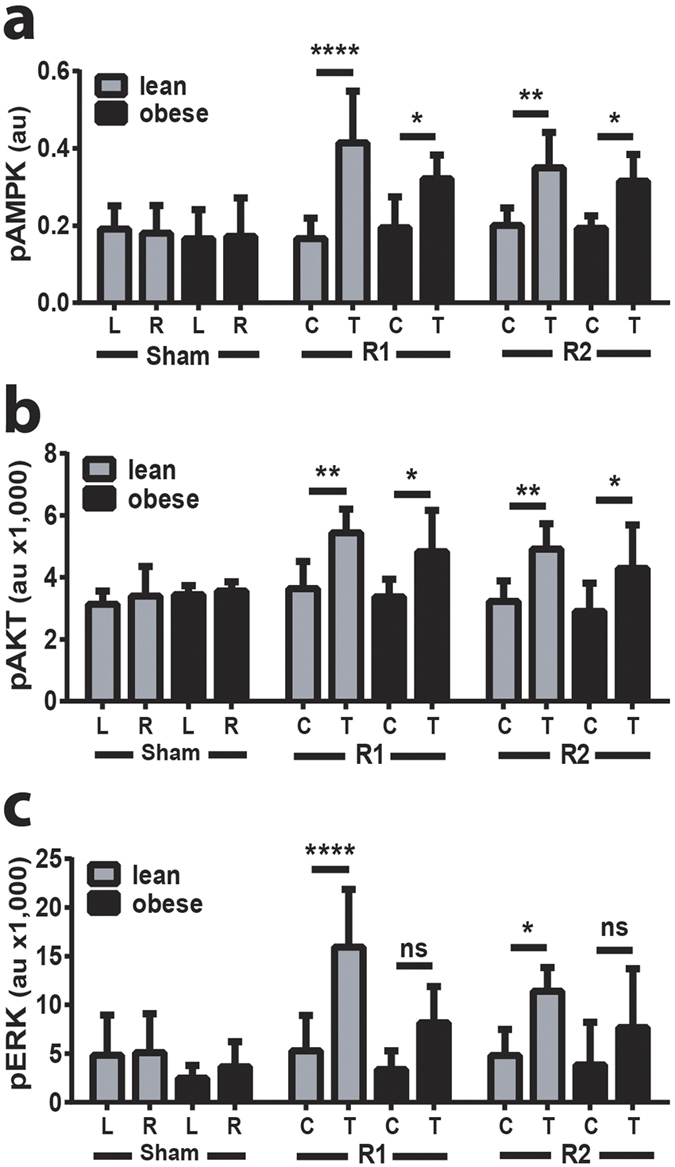
^TAM^R1 or ^TAM^R2 results in increased phosphorylation of downstream signalling effectors. **(a)** Quantitation of AMPK phosphorylation in TAM lysates from lean and obese control (sham), ^TAM^R1 (R1) and ^TAM^R2 (R2) mice determined by ELISA. Quantitation of **(b)** AKT and **(c)** ERK phosphorylation in TAM lysates from sham, R1 and R2 mice determined by Alphascreen. Data are expressed as mean ± SEM; n = 6 in each group; *p < 0.05, **p < 0.01, ***p < 0.001, ****p < 0.0001; significant difference of test (T) vs control (C) TAM in lean or obese mice.

We next sought to examine the local effects of ^TAM^R1 or ^TAM^R2 at the level of proximal phosphorylation events using ELISA or Alphascreen technology. Phosphorylation of AMPK was increased around 2-fold in response to overexpression of either receptor in TAM of lean mice (Fig. 3a). Overexpression of either receptor also significantly increased AMPK phosphorylation in obese mice however the magnitude of this effect was less than that observed in lean mice. Similar results were seen for AKT phosphorylation. ^TAM^R1 or ^TAM^R2 increased AKT phosphorylation in TAM by around 50%, whilst the magnitude of this effect was reduced by around 20% in obese mice (Fig. 3b). In lean mice, phosphorylation of ERK was increased 3-fold by ^TAM^R1 and 2-fold by ^TAM^R2 (Fig. 3c). The magnitude of these effects was reduced by 55% and 45% in obese mice such that overexpression of AdipoR1 or AdipoR2 no-longer resulted in a significant increase in test verses control TAM (Fig. 3c). These changes occurred in the absence of any change in the total levels of AMPK, AKT and ERK proteins (Supplementary Fig. S1). These results show that, at least under these experimental conditions, overexpression of AdipoR1 or AdipoR2 have similar effects on proximal signaling effectors and also suggest the development of HFD-obesity induced adiponectin resistance.

### ^TAM^R2, but not ^TAM^R1, promotes increased expression of genes involved in lipid metabolism and these effects are reduced in HFD-induced obesity

**Figure 4:**
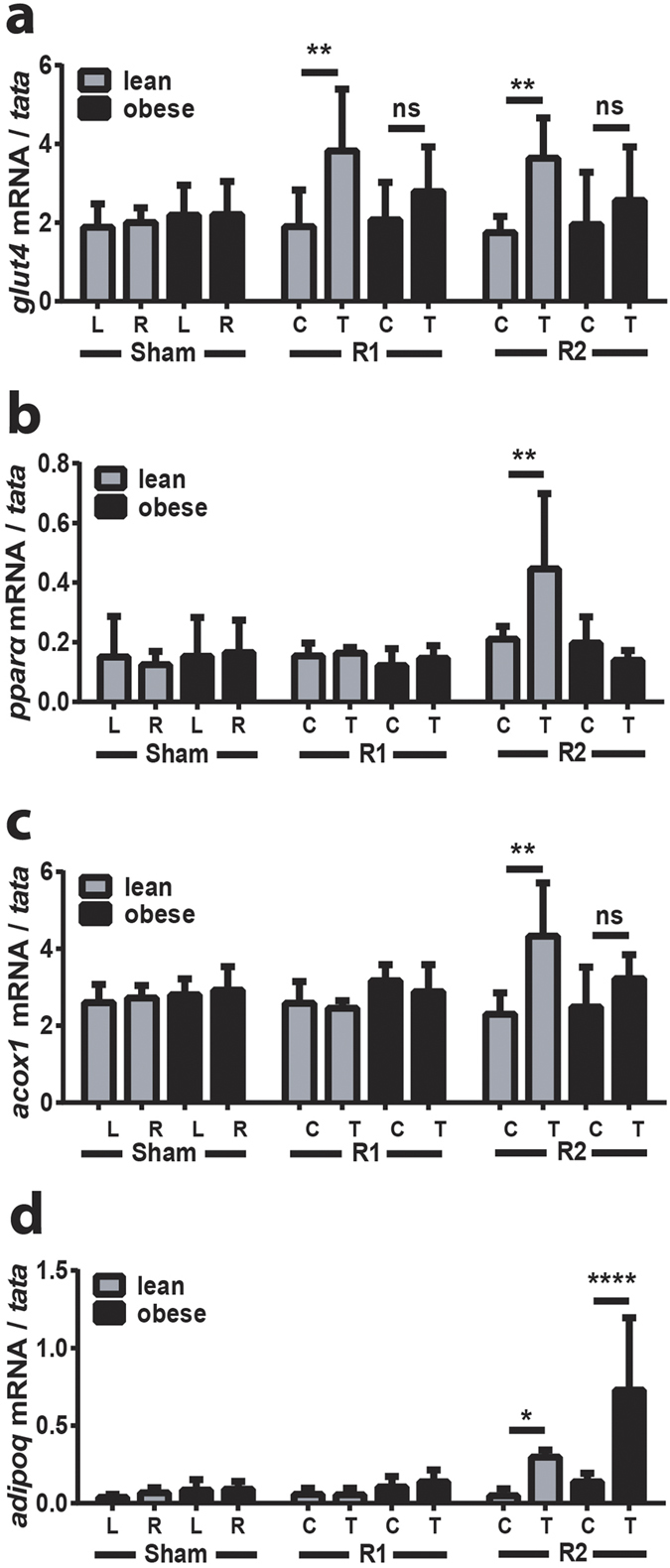
^TAM^R1 or ^TAM^R2 increase *glut4* expression but only the latter increases the expression of *adipoQ* and genes involved in lipid metabolism. qRT-PCR analysis of (**a**) *glut4*, (**b**) *pparα*, (**c**) *acox1* (**d**) and *adipoQ* expression in lean and obese control (sham), ^TAM^R1 (R1) and ^TAM^R2 (R2) mice. Data are expressed as mean ± SEM; n = 6 in each group; *p < 0.05, **p < 0.01, ****p < 0.0001; significant difference of test (T) vs control (C) TAM in lean or obese mice.

We then investigated the effects of overexpression of the receptors on expression of key genes involved in glucose and lipid metabolism in skeletal muscle. Overexpression of AdipoR1 or AdipoR2 resulted in a 2-fold increase in expression of the insulin-responsive glucose transporter *glut4* in lean mice but this effect was not observed in obese mice (Fig. 4a). In contrast to the effects described above that were common to both AdipoR1 and AdipoR2, only the latter effected changes in genes involved in lipid metabolism, *pparα* and a downstream target gene *acox1*. Indeed, in lean but not obese ^TAM^R2 mice *pparα* and *acox1* (encoding acyl-CoA oxidase) were both increased around 2-fold (Fig. 4b and c). It has previously been reported that adiponectin and AdipoR1 promote increased expression of PPARα coactivator 1α (PGC1α) and mitochondrial biogenesis^25^. Therefore, we also examined the effects of overexpression of the receptors on expression of *pgc1α* as well as genes encoding the mitochondrial uncoupling proteins UCP2 and UCP3. However, ^TAM^R1 and ^TAM^R2 had no effect on the expression of these genes in lean or obese mice (Supplementary Fig. S2). These results demonstrate different effects on local gene expression following ^TAM^R1 or ^TAM^R2 and also provide further evidence of adiponectin resistance in the face of HFD-induced obesity.

### ^TAM^R2, but not ^TAM^R1, promotes increased expression of adipoQ in TAM from lean and obese mice

Next, we examined expression of *adipoQ* (encoding adiponectin) in test and control TAM. As expected, expression of *adipoQ* was relatively low in control muscle from lean mice (1000-fold less than in epididymal fat). Obesity increased *adipoQ* expression 2.2-fold (n = 18/group; p = 0.001), consistent with previous reports^26^, whilst ^TAM^R2 increased *adipoQ* levels 6–7 fold in TAM from both lean and obese mice respectively (Fig. 4d).

### ^TAM^R2 in obese mice reduces HFD-induced weight gain, adipose tissue mass and inflammation, and increases circulating adiponectin levels

**Figure 5:**
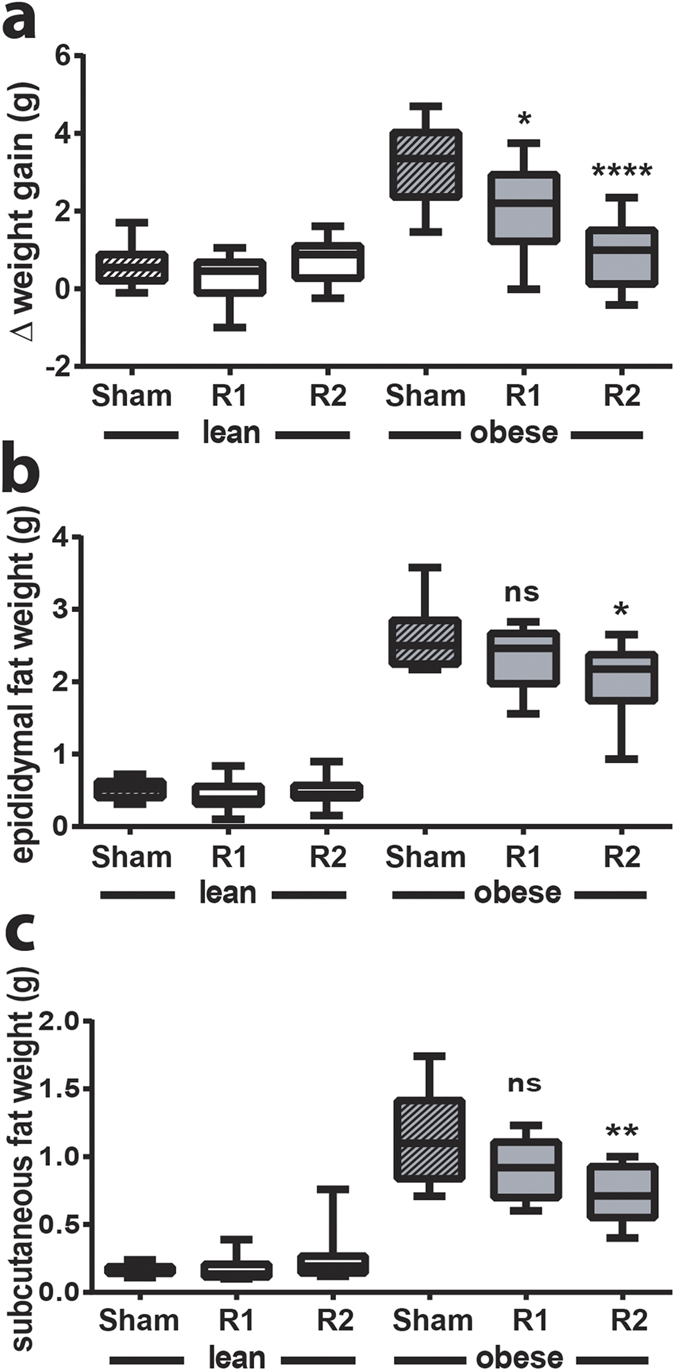
^TAM^R2 reduces weight gain and fat pad weight in obese mice. Graphs showing **(a)** average weight gain and **(b)** epididymal and **(c)** subcutaneous fat pad weights in lean and obese control (sham), ^TAM^R1 (R1) and ^TAM^R2 (R2) mice two weeks post IVE. Data are expressed as mean ± SEM; n = 6 in each group; *p < 0.05, **p < 0.01, ****p < 0.0001; significant difference vs control (sham) obese mice.

**Figure 6:**
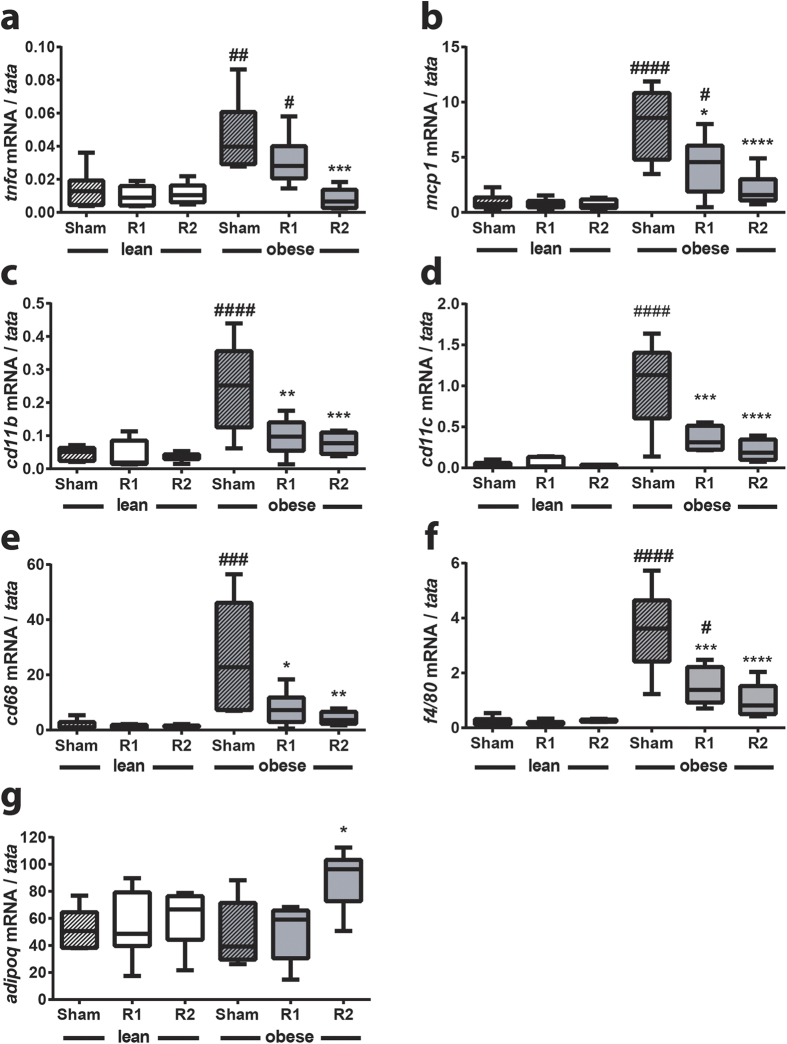
^TAM^R2 reduces HFD-induced inflammation and increases *adipoQ* expression in epididymal fat pads of obese mice. qRT-PCR analysis of (**a**) *tnfα*, (**b**) *mcp1*, (**c**) *cd11b*, (**d**) *cd11c,* (**e**) *cd68*, (**f**) *f4/80* and (**g**) *adipoQ* expression in epididymal fat pads from lean and obese control (sham), ^TAM^R1 (R1) and ^TAM^R2 (R2) mice. Data are presented as mean ± SEM; n = 6 in each group; *p < 0.05, **p < 0.01, ***p < 0.001, ****p < 0.0001; significant difference vs control (sham) obese mice. ^#^p < 0.05, ^##^p < 0.01, ^###^p < 0.001, ^####^p < 0.0001; significant difference between lean and obese mice within genotype (sham, R1 or R2).

**Figure 7:**
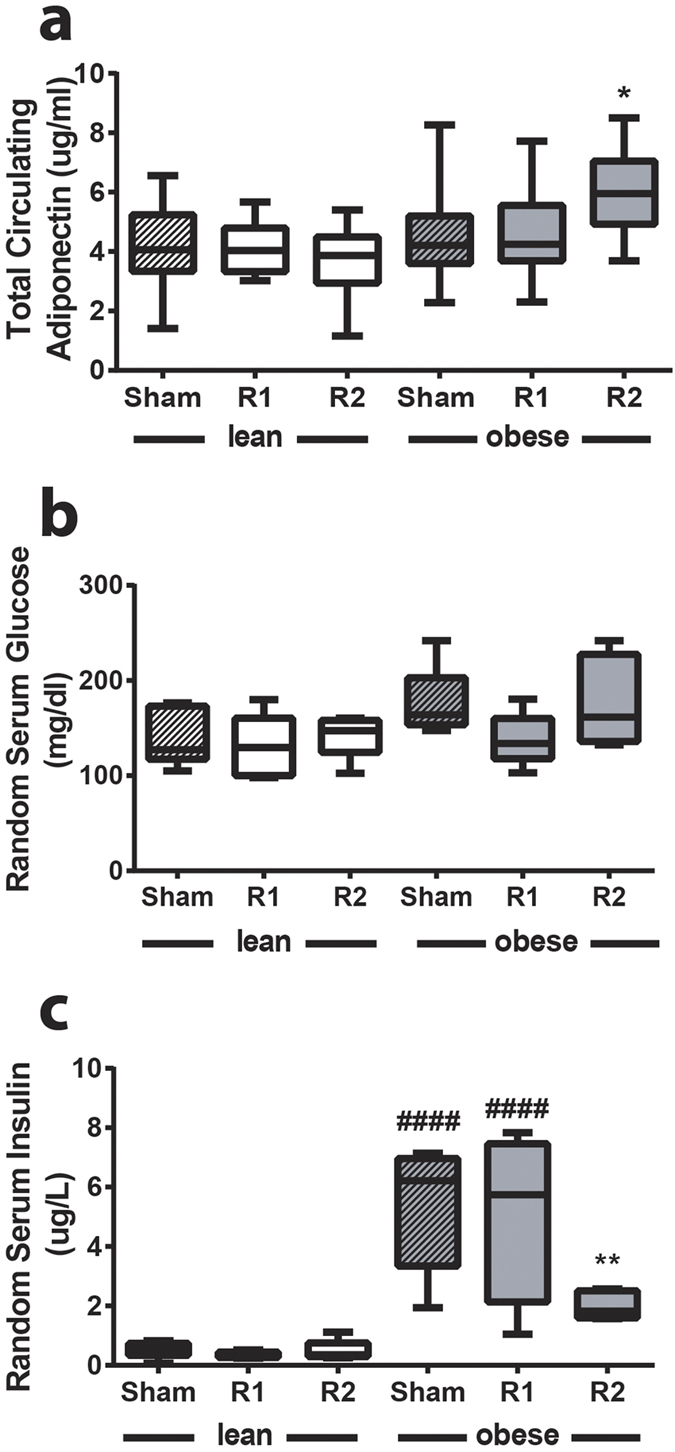
^TAM^R2 increases circulating adiponectin levels and normalises insulin levels in obese mice. Serum levels of **(a)** total adiponectin, **(b)** glucose and **(c)** insulin in non-fasted control (sham), ^TAM^R1 (R1) and ^TAM^R2 (R2) lean and obese mice. Data are presented as mean ± SEM; n = 6 in each group; *p < 0.05, **p < 0.01; significant difference vs control (sham) obese mice. ^####^p < 0.0001; significant difference between lean and obese mice within genotype (sham, R1 or R2).

Consistent with the findings in our pilot study (see above) we again observed a significant decrease in weight gain in ^TAM^R2 obese mice compared with obese control mice (transduced with empty plasmid in both legs) or ^TAM^R1 obese mice (Fig. 5a). The latter also promoted a modest but significant reduction in weight gain compared with the obese control group (Fig. 5a and Supplementary Fig. S3). To address this further we measured epididymal and subcutaneous fat pad weights. Consistent with the reduced weight gain, fat pad weights were also significantly reduced in the ^TAM^R2 obese mice (Fig. 5b and c). In light of these surprising observations we performed qRT-PCR on the epididymal and subcutaneous fat pads (and liver) using primers specific for human *AdipoR2* to rule out the possibility that these effects may reflect leaky expression of AdipoR2 in tissues other than the test TAM. In all cases we were unable to detect human *AdipoR2* expression (data not shown) leaving us to conclude that these effects are most likely mediated indirectly via the increased expression of AdipoR2 in TAM. Having established this, we performed further characterisation of the epididymal fat pads aiming to define the impact on the inflammatory signature. As expected, HFD-induced obesity resulted in a significant increase in expression of inflammatory markers including the pro-inflammatory cytokine TNFα^27^, the chemokine monocyte chemoattractant protein (MCP)−1^28^, the monocyte/macrophage markers CD11b, CD11c, CD68, and F4/80^29^ (Fig. 6a–f). ^TAM^R1 or ^TAM^R2 had no effect on inflammatory gene expression in lean mice. However, in ^TAM^R2 obese mice there was a significant reduction in the expression of all inflammatory markers, such that expression levels were comparable to those in lean mice (Fig. 6a–f). Moreover, ^TAM^R1 obese mice presented an intermediate profile with significant reductions in *mcp1, cd11b, cd11c, cd68* and *f4/80* compared with obese sham mice (Fig. 6a–f). We also determined the effects of diet and gene transduction on *adipoQ* expression. All groups showed similar expression except for the ^TAM^R2 obese mice, where *adipoQ* expression was significantly elevated (Fig. 6g). To investigate this further we measured circulating adiponectin, glucose and insulin levels. Consistent with the gene expression, and our pilot study, circulating adiponectin levels were significantly increased in ^TAM^R2 obese mice (Fig. 7a). Random glucose levels were unaffected by either overexpression of AdipoR1 and AdipoR2 or HFD-induced obesity (Fig. 7b). In contrast, serum insulin levels were markedly elevated in HFD-induced obesity in sham or ^TAM^R1 obese mice (Fig. 7c). This effect was reversed in ^TAM^R2 obese mice (Fig. 7c).

**Figure 8:**
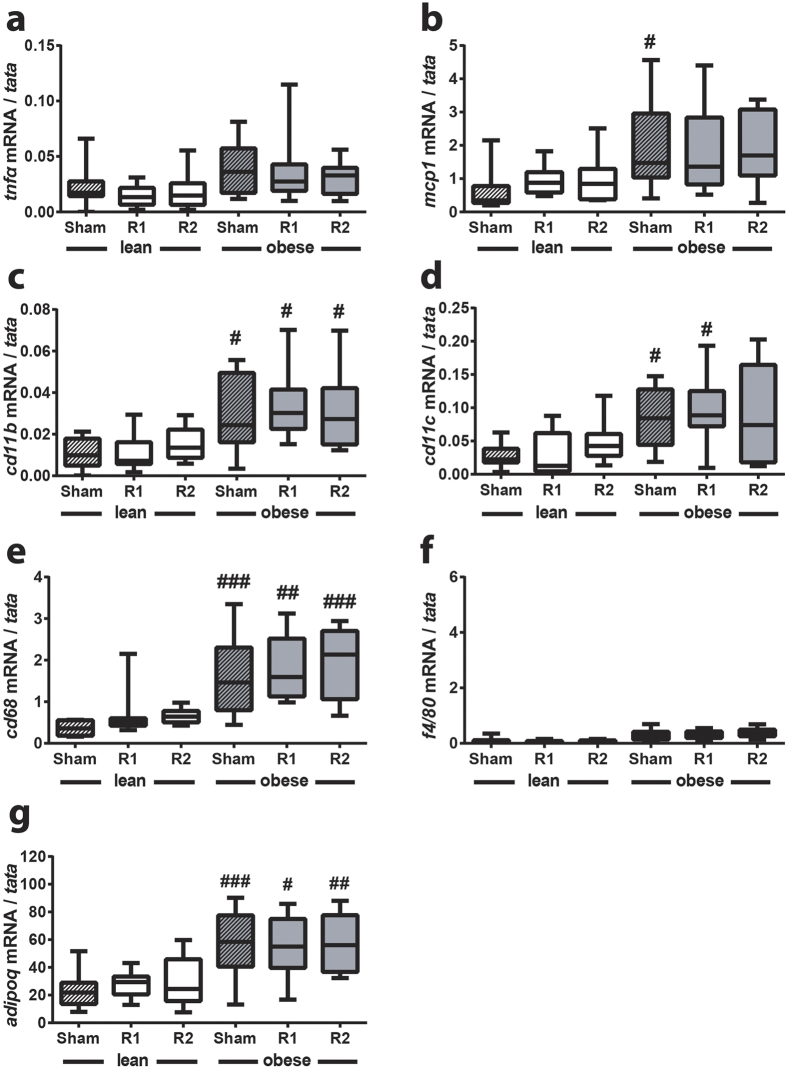
^TAM^R1 or ^TAM^R2 do not affect inflammation in subcutaneous fat pad. qRT-PCR analysis of **(a)** *tnfa*, **(b)** *mcp1*, **(c)** *cd11b*, **(d)** *cd11c,* **(e)** *cd68*, **(e)** *f4/80* and **(g)** *adipoQ* expression in subcutaneous fat pads from lean and obese control (sham), ^TAM^R1 (R1) and ^TAM^R2 (R2) mice. Data are presented as mean ± SEM; n = 6 in each group; ^#^p < 0.05, ^##^p < 0.01, ^###^p < 0.001, ^####^p < 0.0001; significant difference between lean and obese mice within genotype (sham, R1 or R2).

We performed a similar analysis of the subcutaneous adipose tissue which revealed a more modest inflammatory response in the face of HFD-induced obesity and no effect of ^TAM^R1 or ^TAM^R2 (Fig. 8a–f). Surprisingly, *adipoQ* expression was significantly increased by obesity in this depot (Fig. 8g).

Collectively, these results suggest that TAM-specific overexpression of AdipoR2, and to a lesser extent AdipoR1, results in reduced HFD-induced weight gain concomitant with amelioration of HFD-induced adipose inflammation in epididymal fat pads, increased adiponectin expression and circulating adiponectin levels as well as reduced hyperinsulinemia.

## Discussion

In the current study we aimed to extend molecular and cellular studies by comparing the effects of overexpression of AdipoR1 or AdipoR2 in skeletal muscle in lean and obese mice. We employed IVE to mediate overexpression of AdipoR1 or AdipoR2 in TAM of lean or HFD-induced obese mice. In lean mice TAM-specific overexpression of either receptor resulted in increased phosphorylation of downstream effectors and elevated expression of the insulin responsive glucose transporter *glut4*. In contrast, only overexpression of AdipoR2 resulted in increased expression of *pparα* and the target gene *acox1*. In obese mice the magnitude of all of these effects was reduced even though receptor expression and circulating adiponectin levels were maintained or increased. Surprisingly, ^TAM^R2 in obese mice resulted in a significant decrease in weight gain, adipose tissue mass and inflammation and a significant increase in circulating adiponectin levels. Collectively, these results identify overlapping effects of AdipoR1 and AdipoR2 as well as additional, distinct effects of the latter that provide a foundation for further investigations aimed at reducing obesity-related complications.

Investigations at the molecular and cellular level have provided clear evidence that AdipoR1 and AdipoR2 display different properties in terms of adiponectin binding^12^, cell surface expression, oligomerization and signaling^17^,^19^,^20^,^30^. Consistent with this scenario, whole animal studies, predominantly involving characterization of AdipoR1 or AdipoR2 knockout mice, have demonstrated different signaling outputs, such as coupling of hepatic AMPK and PPARα activity to AdipoR1 and AdipoR2 respectively^21^, and phenotypic consequences following deletion of either receptor^21^,^31^,^32^,^33^. Whilst informative, there are caveats to this loss of function approach given oligomerization of AdipoR1 and AdipoR2^12^,^19^,^34^ has been shown to alter properties of the receptor complex and downstream signaling outputs^17^,^19^. For example, our observations that under physiological conditions (no serum withdrawal) cell surface expression of AdipoR2 is limited unless it is co-expressed with AdipoR1^17^ has clear implications when considering the impact of AdipoR1 deletion, which is also likely to compromise AdipoR2 function. Furthermore, adiponectin receptor interacting proteins such as ERp46 have been shown to modulate receptor oligomerization, cell surface expression and downstream signaling^24^. Thus, a gain of function approach may be expected to provide important complementary information. Typically such studies have tended to focus on the effects of overexpression of either AdipoR1 or AdipoR2. For example, overexpression of AdipoR1 in rat skeletal muscle was reported to improve insulin sensitivity^23^ whilst overexpression of AdipoR2 in liver increased PPARα and protected against progression of NASH^35^. To the best of our knowledge there are no examples where such studies have compared the effects of overexpression of AdipoR1 or AdipoR2 in skeletal muscle *in vivo*.

Skeletal muscle is a recognized target of adiponectin action, with adiponectin increasing fatty acid oxidation and glucose uptake and enhancing insulin sensitivity by activation of pathways involving AMPK and PPARα^1^,^26^,^36^. In addition, numerous studies have provided evidence of adiponectin resistance in skeletal muscle from rodents^5^,^7^,^8^,^37^ and humans^3^,^4^,^9^. Whilst muscle-specific deletion of AdipoR1 has established a key role for AdipoR1^25^ this does not preclude a role for AdipoR2 in mediating the beneficial effects of adiponectin in skeletal muscle or the potential of AdipoR2-based therapies. Thus, in the current study we used IVE to compare the effects of overexpression of AdipoR1 or AdipoR2 in TAM of lean or HFD-induced obese mice.

Neither IVE nor HFD-induced obesity affected expression of endogenous receptors at the mRNA level and endogenous *adipoR1* was expressed around 10-fold higher than *adipoR2,* consistent with a previous report^12^. Measurement of both gene and protein (HA) indicated that the exogenous human receptors were expressed at similar levels, with IVE increasing total AdipoR2 levels in TAM by around 100%. Unfortunately we were unable to determine the impact of AdipoR1 overexpression on total levels due to the lack of a suitable antibody. Nevertheless, similar levels of overexpression of AdipoR1 and AdipoR2 in TAM of lean mice resulted in comparable elevation of phosphorylation events including AMPK, AKT and ERK as well as increased *glut4* expression suggesting that, at least under these conditions, they mediate similar effects consistent with enhanced adiponectin and insulin sensitivity. Furthermore, these beneficial effects were reduced in the context of HFD-induced obesity by 20–60%. Given circulating adiponectin levels were not decreased these findings are consistent with the development of adiponectin resistance, at a level distal to receptor expression.

Only overexpression of AdipoR2 resulted in increased expression of *pparα* and *acox1*. This is consistent with the findings of impaired hepatic PPARα activity in AdipoR2 knockout mice^21^ but contrasts with observations in endothelial cells, where overexpression of either AdipoR1 or AdipoR2 was sufficient to mediate PPARα activation^38^. Once again, these effects were blunted in obesity providing further evidence of adiponectin resistance.

Emerging evidence suggests adiponectin is produced by skeletal muscle and that this is increased in response to obesity or inflammation^26^,^39^,^40^. Consistent with these observations, we observed a 2-fold increase in *adipoQ* levels in TAM from obese mice. Intriguingly, overexpression of AdipoR2 promoted increased expression of *adipoQ* in both lean and obese mice. The molecular basis for this effect is unclear, particularly given that all other local effects of ^TAM^R2 were diminished in obese mice. Further investigations are warranted to elaborate the underlying mechanisms, which may reveal novel strategies to induce adiponectin expression more globally.

Perhaps the most surprising observations in this study relate to the effects of ^TAM^R2 reducing weight gain in obese mice. Indeed, weight gain in HFD-fed obese mice transduced with AdipoR2 was indistinguishable from that in the lean, chow-fed mice. This effect was also reflected by a modest but significant reduction in epididymal and subcutaneous fat pad weights, compared with those from obese sham or ^TAM^R1 mice, and a striking resolution of adipose tissue inflammation in the epididymal fat pad. Moreover, *adipoQ* expression was significantly elevated in the epididymal fat pad from the obese ^TAM^R2 mice as were circulating levels of adiponectin. It is noteworthy that these observations (reduced weight gain and elevated adiponectin) are consistent across two independent studies, performed in two distinct research facilities, using different sets of reagents with different mouse cohorts. Whilst unexpected, evidence from the literature supports the notion that overexpression of either AdipoR1 or AdipoR2 may prevent weight gain. Hydrodynamic delivery of AdipoR2 to the liver resulted in reduced diet-induced weight gain and adipose tissue mass^41^ whilst global or macrophage-specific overexpression of AdipoR1 were also sufficient to reduce diet-induced weight gain^42^,^43^. Whilst it remains possible that the IVE approach employed in the current study may have resulted in transduction of cells other than the TAM we were unable to detect evidence of such leaky expression in epididymal or subcutaneous fat pads or in liver. Thus, we propose that overexpression of AdipoR2 in skeletal muscle results in altered expression of a circulating factor, possibly a myokine in a manner similar to that detailed for *adipoQ*, and that this underpins the reduced weight gain and associated improvements. It also remains possible that the observed changes may be due, at least in part, to alterations in locomotor activity. Clearly, further studies are required to investigate this intriguing hypothesis.

In summary, we have demonstrated that overexpression of AdipoR1 or AdipoR2 in mouse skeletal muscle promote similar effects at the level of proximal signaling events and *glut4* expression whilst only AdipoR2 promotes activation of the PPARα axis. All of these effects were blunted in the face of obesity, consistent with the development of adiponectin resistance at the level of skeletal muscle. Finally, muscle-specific overexpression of AdipoR2 gave rise to several unexpected local and systemic effects that included increased expression of *adipoQ* in muscle and epididymal adipose as well as increased circulating levels of adiponectin, and reduced HFD-induced weight gain, adipose tissue mass and inflammation, and reduced hyperinsulinemia. However, these effects appeared unable to ameliorate muscle adiponectin resistance. Future studies, investigating the effects of more global muscle-specific overexpression of AdipoR2 may provide further insights into the underlying mechanisms which may provide novel strategies to reverse hypoadiponectinemia and or adiponectin resistance.

## Materials and Methods

### Animals

All experimental procedures were carried out in accordance with approved protocols by the Alfred Medical Research Education Precinct (AMREP) Animal Ethics Committee or the University of Queensland Animal Ethics Unit and were conducted using the Guidelines of the National Health and Medical Research Council. Male WT C57Bl/6 mice (AMREP AS, Melbourne, VIC, Australia or University of Queensland Biological Resource, Brisbane, QLD, Australia) were used for all experiments and commenced when mice were 8 weeks of age. Animals were maintained at 22.0 ± 0.5 °C under a 12-h day, 12-h night cycle and fed standard chow diet containing 5% of total energy from fat. At 8 weeks of age, animals were divided into two groups. Half kept on standard chow diet and the rest were fed high fat diet containing 43% of total calories from fat (lard) (Specialty Feeds, Glen Forrest, WA, Australia) for 8 weeks before *in vivo* electrotransfer.

### Reagents and antibodies

Reagents were from Sigma–Aldrich (Castle Hill, Australia) unless otherwise stated. Primary antibodies against HA and Sodium Potassium ATPase were from Covance (Washington, USA) and Abcam (Melbourne, Australia) respectively. Primary antibodies against AMPK, AKT and ERK were from Cell-Signaling technology (Danvers, Massachusetts). ‘In-house’ AdipoR2 antibody has been described previously^24^. Secondary antibodies were from Life Technology (Invitrogen).

### Molecular biology

Original constructs encoding C-terminally epitope-tagged (HA) human AdipoR1 and AdipoR2 were as described^24^. Plasmid DNA was prepared using a Plasmid Purification Gigaprep kit (Qiagen, Valencia, CA, USA) according to the manufacturer’s specifications. The DNA concentration was quantified using a Nanodrop ND-1000 Spectrophotometer (Biolab, Scoresby, VIC, Australia) and the DNA dissolved in saline (154 mmol/l NaCl) to a final concentration of 4 μg/μl.

### *In vivo* electrotransfer (IVE)

IVE was performed as described^22^. Briefly, mice were anaesthetised with isoflurane, their hind limbs were shaved and TA muscles were injected with 30 μl of 0.5 U/μl hyaluronidase. Two hours later mice were again anaesthetised with isoflurane and 100 μg empty vector, GFP, HA-tagged AdipoR1 or HA-tagged AdipoR2 (in 25 μl saline) was injected into the right TA muscle. The left TA muscle was injected with 25 μl empty vector. Stainless steel electrodes attached to an ECM-830 electroporator (BTX, Holliston, MA) were placed on the muscle and square-wave electrical pulses (200 V/cm) were applied eight times with an electrical pulse generator at a rate of 1 pulse/s, with each pulse being of 20 ms duration. Two weeks later muscles and other tissues were dissected, snap frozen in liquid nitrogen and stored at −80 °C.

### Real-time PCR

Gene expression levels were quantified using real-time PCR assay. Mice TAMs were powdered under liquid nitrogen and homogenized using Fast prep-24 (MP Biomedicals, NSW, Australia) in Trizol (Invitrogen, Australia). Epididymal and subcutaneous adipose tissue and liver were homogenized as above. Total RNA was then extracted as per the manufacturer’s instructions and resuspended in nuclease-free water. Genomic DNA contamination of RNA preparations was eliminated by digestion with DNase I amplification grade (Invitrogen, Australia). cDNA was synthesized from 1 µg total RNA using cDNA synthesis kit (Bioline, NSW, Australia) and RT-PCR was performed using the SYBR Hi-ROX kit (Bioline) on a 7900HT Fast Real-time PCR system (Ambion Life Technologies). Results are quoted to the mRNA level compared to TATA box, the expression of which was unchanged by the treatments. Primer sequences are listed in Supplementary Table S1.

### Western blot

Mice TAMs were powdered under liquid nitrogen and homogenized using Fast prep-24 (MP Biomedicals, NSW, Australia) in lysis buffer. Lysates were depleted of nuclei via centrifugation at 800 g for 10 min. The supernatant was then centrifuged at 257,000 g for 1 h, and the pellet (HSP containing membrane) was resuspended in lysis buffer and protein concentration was determined by bicinchononic acid (BCA) assay (Pierce) using BSA as the standard. 60 μg protein was then loaded and resolved by SDS-PAGE on polyacrylamide gels, transferred to membranes and blocked with 5% BSA. The immunoreactive proteins were detected with enhanced chemiluminescence after incubation with appropriate primary and secondary antibodies.

### Measurement of circulating adiponectin, glucose and insulin levels

All analyses were made on serum taken from non-fasted animals and were performed in accordance with the manufacturer’s instructions. Total circulating adiponectin was measured using an adiponectin ELISA (R&D Systems, Minneapolis, MN, USA). Blood glucose was measured using a glucose colorimetric assay kit (Cayman chemical, Michigan, USA). Insulin levels were measured using a mouse insulin ELISA (Mercodia, Uppsala, Sweden).

### AMPK phosphorylation ELISA

TAMs were powdered under liquid nitrogen and homogenized using Fast prep-24 (MP Biomedicals, NSW, Australia) in lysis buffer. Phosphorylation of AMPK in TA muscle was measured using a PathScan Phospho-AMPKα (Thr172) Sandwich ELISA (Cell-Signaling technology, Danvers, Massachusetts) according to the manufacturer’s instruction.

### Akt and ERK phosphorylation assays

Alphascreen analysis was performed as previously described^20^. TAMs were powdered under liquid nitrogen and homogenized using Fast prep-24 (MP Biomedicals, NSW, Australia) in Alphascreen lysis buffer. Phosphorylation of Akt and ERK was measured using AlphaScreen SureFire kits essentially as described (PerkinElmer Life and Analytical Sciences, Waltham, MA, USA). Plates were read using a POLARstar Omega plate reader.

### Statistical analysis

Data are presented as mean ± SEM. Significance was determined using paired t test to compare control and test TAM and two-way ANOVA followed by Tukey’s multiple comparison test with statistical significance defined as p < 0.05.

## Additional Information

**How to cite this article**: Keshvari, S. *et al*. Muscle-specific overexpression of AdipoR1 or AdipoR2 gives rise to common and discrete local effects whilst AdipoR2 promotes additional systemic effects. *Sci. Rep.* **7**, 41792; doi: 10.1038/srep41792 (2017).

**Publisher's note:** Springer Nature remains neutral with regard to jurisdictional claims in published maps and institutional affiliations.

## Supplementary Material

Supplementary Information
