# Supplementary material for: Muscle-specific overexpression of AdipoR1 or AdipoR2 gives rise to common and discrete local effects whilst AdipoR2 promotes additional systemic effects
Source: Sci Rep. 2017 Feb 1;7:41792. doi: 10.1038/srep41792 (PMC5286438; doi:10.1038/srep41792)
Supplement: Supplementary Information [file srep41792-s1.pdf]

Supplementary Information

Muscle-specific overexpression of AdipoR1 or AdipoR2 gives rise to common and discrete local effects whilst AdipoR2 promotes additional systemic effects.

Sahar Keshvari[1], Darren C. Henstridge[2], Choaping Ng[1], Mark A. Febbraio[2,3], Jonathan P. Whitehead[1*]

[1] University of Queensland, Mater Research Institute-UQ, Brisbane, QLD 4102, Australia.

[2] Cellular and Molecular Metabolism Laboratory, Baker IDI Heart and Diabetes Institute, Melbourne, VIC 3004, Australia.

[3] Division of Diabetes & Metabolism, Garvan Institute of Medical Research, Darlinghurst, NSW 2010, Australia.

* Corresponding author: Translational Research Institute, Mater Research Institute-UQ, Brisbane, QLD 4102, Australia. Tel.: +61 07 3443 7633; fax: +61 0 7 3443 7779.

E-mail address: jon.whitehead@mater.uq.edu.au (J.P. Whitehead).

Supplementary Figure Legends:

Supplementary Fig S1. ^{TAM}R1 or ^{TAM}R2 do not affect total AMPK, AKT and ERK protein levels in TAM. Western blot analysis of cytosolic (soluble) fraction of TAM lysates derived from left (L) and right (R) leg of control, ^{TAM}R1 and ^{TAM}R2 mice probed with **(a)** AMPK, **(b)** AKT and **(c)** ERK antibodies; n=3 in each group of lean and obese mice.

Supplementary Fig S2. ^{TAM}R1 or ^{TAM}R2 do not affect mitochondrial marker expression in TAM. qRT-PCR analysis of **(a)** *ucp2* **(b)** *ucp3* and **(c)** *pgc1α* expression in control (sham), ^{TAM}R1 (R1) and ^{TAM}R2 (R2) mice. Data are expressed as mean ± SEM; n=6 in each group.

Supplementary Fig S3. ^{TAM}R2 improves HFD induced weight gain. Average body weight of mice fed either a chow or high-fat diet for 10 weeks. *In vivo* electrotransfer (IVE) was performed at eight weeks of diet intervention. Values are presented as mean±SEM; n=6 in each group.

Supplementary Table S1. Primer sequences used for qRT-PCR.

Supplementary Fig S1

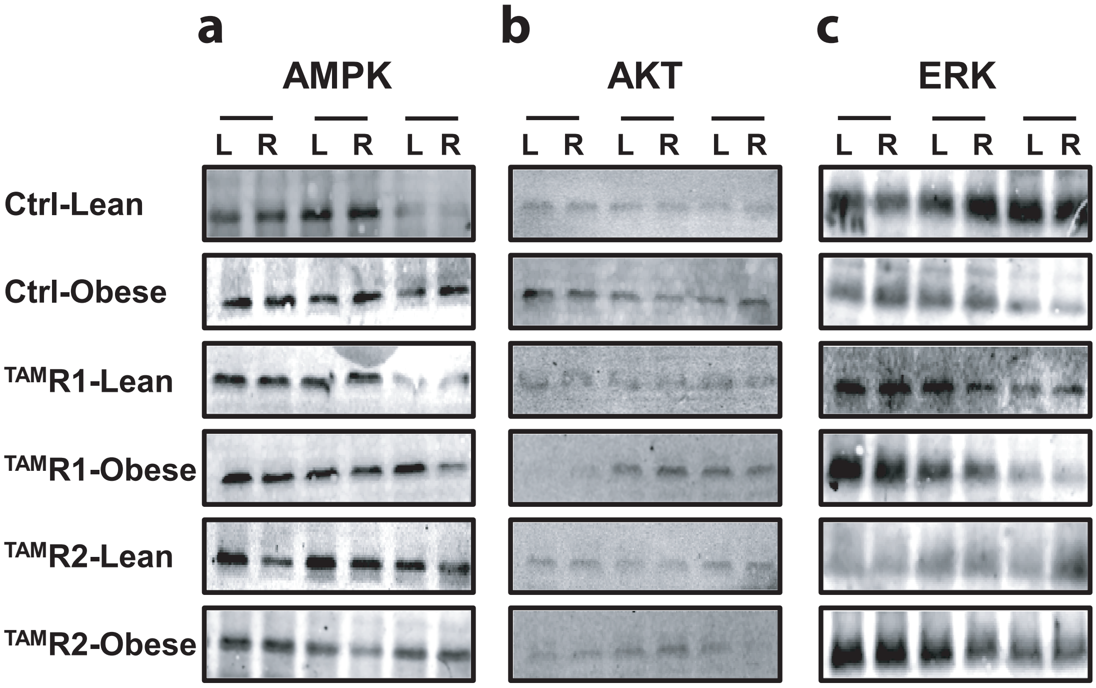

Supplementary Fig S2

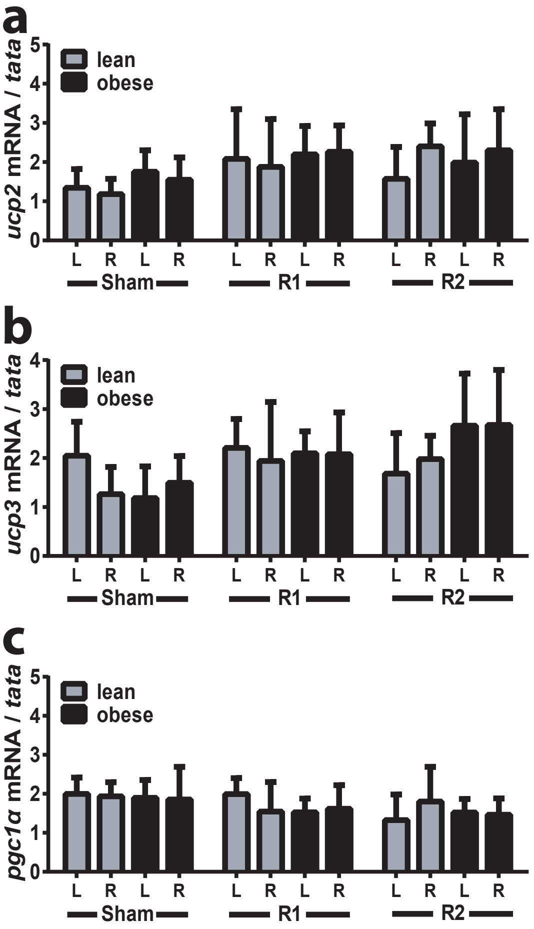

Supplementary Fig S3

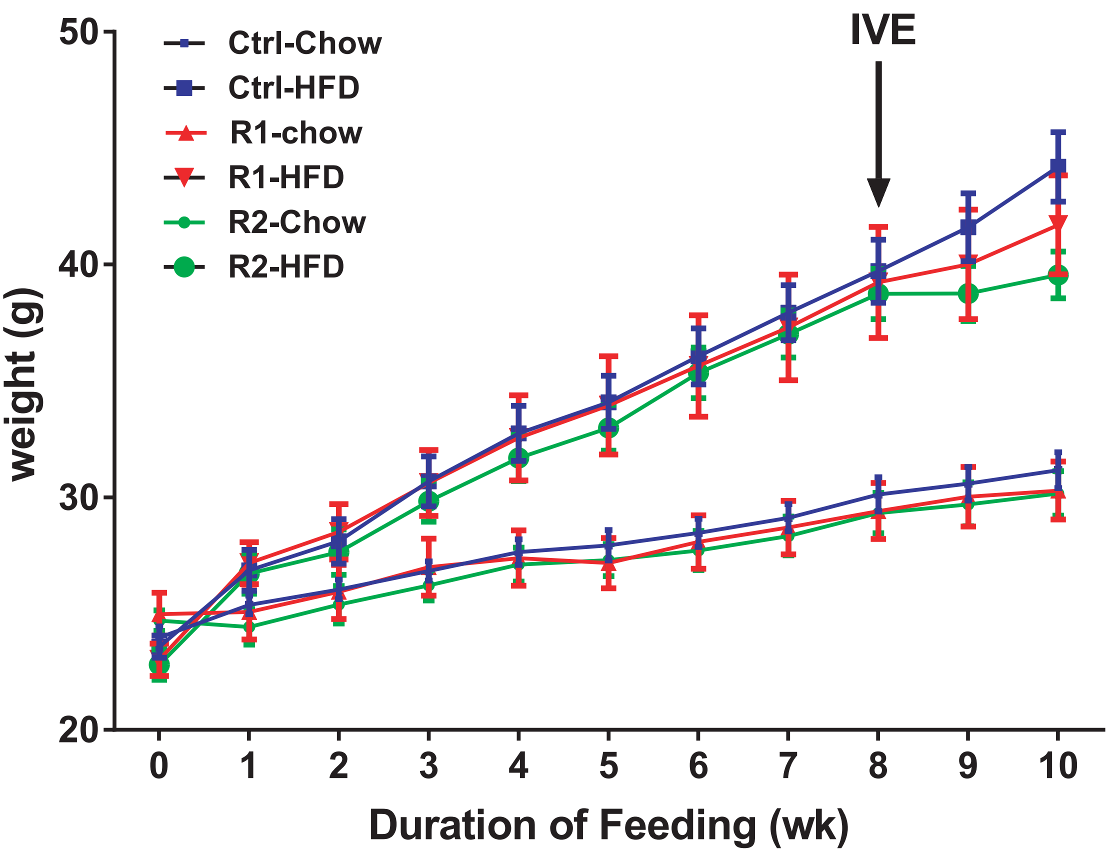

Supplementary Table S1

primer	sequence 5´→ 3´
R1-HA	F: AATTCCTGAGCGCTTCTTTCC
	R: GTAATCTGGCACATCGTATGG
R2-HA	F: GATCCCCGAACGCTTTTTCC
	R: GTAATCTGGCACATCGTATGG
mouse R1	F: TCTGCCTCAGTTTCTCCTGGCT
	R: GTAATAGAGCCAGGGAACGAAGC
mouse R2	F: TCTTCCACACGGTGTACTGCCA
	R: GGTAGATGAAGCAAGGTTGTGGG
mouse adipoQ	F: AGATGGCACTCCTGGAGAGAAG
	R: ACATAAGCGGCTTCTCCAGGCT
mouse GLUT4	F: GGTGTGGTCAATACGGTCTTCAC
	R: AGCAGAGCCACGGTCATCAAGA
mouse PPARα	F: ACCACTACGGAGTTCACGCATG
	R: GAATCTTGCAGCTCCGATCACAC
mouse Acox1	F: GCCATTCGATACAGTGCTGTGAG
	R: CCGAGAAAGTGGAAGGCATAGG
mouse ACC2	F: AGAAGCGAGCACTGCAAGGTTG
	R: GGAAGATGGACTCCACCTGGTT
mouse TNFα	F: GGTGCCTATGTCTCAGCCTCTT
	R: GCCATAGAACTGATGAGAGGGAG
mouse MCP1	F: GCTACAAGAGGATCACCAGCAG
	R: GTCTGGACCCATTCCTTCTTGG
mouse Cd11b	F: TACTTCGGGCAGTCTCTGAGTG
	R: ATGGTTGCCTCCAGTCTCAGCA
mouse Cd11c	F: TGCCAGGATGACCTTAGTGTCG
	R: CAGAGTGACTGTGGTTCCGTAG
mouse Cd68	F: GGCGGTGGAATACAATGTGTCC
	R: GGCGGTGGAATACAATGTGTCC
mouse F4-80	F: CGTGTTGTTGGTGGCACTGTGA
	R: CCACATCAGTGTTCCAGGAGAC
mouse IL-10	F: AGCTCCAAGACCAAGGTGTC
	R: TCCAAGGAGTTGTTTCCGTTA
mouse UCP2	F: TAAAGGTCCGCTTCCAGGCTCA
	R: ACGGGCAACATTGGGAGAAGTC
mouse UCP3	F: CAACTGTGCTGAGATGGTGACC
	R: TGGCACAGAAGCCAGCTCCAAA
mouse PGC1a	F: GAATCAAGCCACTACAGACACCG
	R: CATCCCTCTTGAGCCTTTCGTG
GFP	F: AAGCTGACCCTGAAGTTCATCTGC
	R: CTTGTAGTTGCCGTCGTCCTTGAA
mouse TATA box	F: CTCAGTTACAGGTGGCAGCA
	R: ACCAACAATCACCAACAGCA
